# Supplementary material for: PLA2R antibody, PLA2R rs4664308 polymorphism and PLA2R mRNA levels in Tunisian patients with primary membranous nephritis
Source: PLoS One. 2020 Oct 1;15(10):e0240025. doi: 10.1371/journal.pone.0240025 (PMC7529277; doi:10.1371/journal.pone.0240025)
Supplement: S1 File — This document describes the consent that was signed by each patient included in this study. (PDF) [file pone.0240025.s001.pdf]

Ministère de la santé publique
Hôpital Charles Nicolle
Laboratoire d'immunologie
Pr. Yousr GORGI

Ministère de la santé publique
Hôpital Charles Nicolle
Service de Néphrologie (M8)
Pr. Taieb Ben Abdallah

Formulaire de consentement

Pour la participation à

L'étude immunogénétique, sérologique et fonctionnelle du PLA2R au cours de la glomérulonéphrite extramembraneuse primitive

De Mr/Mme .... (nom, prénom)

Le Dr. .... m'a proposé(e) de participer à la recherche organisée par les Docteurs Yousr Gorgi et Taieb Ben Abdallah sur la polyarthrite rhumatoïde. Ce projet de recherche est intitulé : « étude immunogénétique, sérologique et fonctionnelle du PLA2R au cours de la glomérulonéphrite extramembraneuse primitive ».

Afin d'éclairer ma décision, j'ai reçu et bien compris les informations suivantes :

Je suis atteint(e) de glomérulonéphrite extramembraneuse primitive. Cette maladie multifactorielle possède une composante génétique multigénique complexe incluant l'implication du gène du PLA2R avec la présence d'anticorps anti-PLA2R. Le dosage des anticorps anti-PLA2R possède un intérêt diagnostique et pronostique

La durée de ma participation à l'étude correspond à celle nécessaire à la réalisation d'un seul prélèvement sanguin (environ 10 ml).

Les analyses moléculaires visant à identifier l'impact du polymorphisme rs4664308 du PLA2R dans la survenue de cette maladie seront effectuées in vitro sur les acides nucléiques extraits des cellules sanguines.

La quantification de l'expression rénale du PLA2R circulante sera réalisé à partir de la même biopsie ayant servi au diagnostic.

Toutes les données recueillies et tous les résultats obtenus dans cette étude resteront strictement confidentiels et ne seront publiés, le cas échéant, dans des revues médicales que sous le sceau strict de l'anonymat. Je n'autorise leur consultation que par des personnes mandatées par les responsables de cette étude, organisateurs de la recherche, pour collaborer à celle-ci et, éventuellement, par un représentant des Autorités de Santé.

Je peux à tout moment demander toute information complémentaire auprès du Docteur .... (n°de tel. : ....).

Après en avoir discuté et avoir obtenu réponse à toutes mes questions, j'accepte librement et volontairement de participer à la recherche décrite ci-dessus. Je suis parfaitement conscient que je peux retirer à tout moment mon consentement à ma participation à cette recherche et cela quelles que soient mes raisons et sans supporter aucune responsabilité. Le fait de ne plus participer à cette recherche ne portera pas atteinte à mes relations avec le médecin investigateur.

Le médecin m'a précisé que je suis libre d'accepter ou de refuser ; cela ne modifiera en rien, ni la prise en charge thérapeutique, ni les relations de confiance mutuelles établies entre moi-même et l'équipe soignante.

Mon consentement ne décharge en rien les organisateurs de la recherche de leurs responsabilités, et je conserve tous mes droits garantis par la loi.

Fait à ………………, le ………………

Signature

Signature de l'investigateur

Un exemplaire cosigné doit être remis à la personne qui participe à la recherche
