# Supplementary material for: PLA2R antibody, PLA2R rs4664308 polymorphism and PLA2R mRNA levels in Tunisian patients with primary membranous nephritis
Source: PLoS One. 2020 Oct 1;15(10):e0240025. doi: 10.1371/journal.pone.0240025 (PMC7529277; doi:10.1371/journal.pone.0240025)
Supplement: Attachment — Submitted filename: Response to reviewers.doc [file pone.0240025.s004.doc]

**Letter to editor**

**List of changes**

**Revision point by point of the manuscript:** **PLA2R antibody, PLA2R rs4664308 polymorphism and PLA2R mRNA levels in Tunisian patients with primary membranous nephritis**

Dear editor,

As required, revision of our article “PLA2R antibody, PLA2R rs4664308 polymorphism and PLA2R mRNA levels in Tunisian patients with primary membranous nephritis” was made. All changes made are **in red color** in the “Revised manuscript with track changes”. We will provide here a point-by-point response to reviewers’ comments:

**Journal Requirements**

1. We ensured that the manuscript meets PLOS ONE’s style requirements.

2. We are reporting three types of standard analyzes: a) an ELISA for anti-PLA2R antibody measurement, b) a real-time PCR for PLA2R rs4664308 polymorphism genotyping and c) For the renal PLA2R expression we used a commercial kit for RNA extraction, then we performed a real-time PCR using the TaqMan gene expression tests Hs00234853_m1 (PLA2R) and Hs00922492_m1 (Podocin), **which were previously used in the study of Hoxha E, et al. Enhanced expression of the M-type phospholipase A2 receptor in glomeruli correlates with serum receptor antibodies in primary membranous nephropathy. Kidney Int 2012; 82(7): 797-804 doi: 10.1038/ki.2012.209**. We added in the methods section the link to the rs4664308 SNP sequence data (<https://www.ncbi.nlm.nih.gov/snp/rs4664308>).

3. We provided the Methods section with the recruitment date range, inclusion and exclusion criteria for PMN patients and demographic details for all participants.

4. Funding information was deleted from the manuscript and was updated online. We confirm that the following Financial Disclosure statement is accurate:

This study was supported by the Research Laboratory in Immunology of Renal Transplantation and Immunopathology (LR03SP01), and the Research Laboratory of Kidney Diseases (LR00SP01), Charles Nicolle Hospital, Tunis El Manar University, Tunisia.

5. The ethics statement is in the **subjects**’ section within the **Material and Methods** chapter.

**Comments to the Author**

**Review comments to the author**

**Reviewer #1:**

**Major comments**

Comment 1: We provided the abstract with information on techniques used and we specified that PMN patients were analyzed for renal PLA2R expression. “increased” was replaced by “higher”. The fold factor cannot be obtained as we used the Kruskal-Wallis test which provided the analysis only with **average ranks** as follow: Complete remission = 17.9, Partial remission = 39.93 and Non-remission = 56.88. The choice of the Kruskal-Wallis test was dictated by the non-Gaussian distribution of the renal PLA2R expression.

Comment 2: In the revised manuscript, from out of the 8 references cited in the introduction section, 5 are original works [3, 5-8]. We did not intend to link Treg decrease to PLA2R expression but to state that both can trigger anti-PLA2R antibody production. Nevertheless, some authors hypothesized that a constant low level of soluble PLA2R maintains peripheral tolerance by controlling Treg function which inhibits anti-PLA2R antibody production by autoreactive B cells [Van de Logt AE, Fresquet M, Wetzels JF, Brenchley P. The anti-PLA2R antibody in membranous nephropathy: what we know and what remains a decade after its discovery. Kidney Int 2019; 96(6): 1292-1302. doi: 10.1016/j.kint.2019.07.014]. In order to make the idea clearer we separated it in two distinct sentences.

Comment 3: We agree that the autoimmune phase could be triggered outside the kidney and we modified the introduction and the discussion sections as recommended by the reviewer #1. In the revised manuscript, we replaced “The current pathophysiological hypothesis stipulates” by “Some authors hypothesized that” at the beginning of the paragraph. We also added that conformational changes and/or increased expression of PLA2R **outside** the kidney could induce anti-PLA2R antibody production, and in this regard the lungs might be relevant. Both ideas are now supported with appropriate citation.

Comment 4: The study of “Hoxha E, et al” is now cited in the introduction section.

Comment 5: The treatment regimen is now recorded in Table 1 and in a new paragraph in subjects’ section. The treatment of PMN patients was based on the KDIGO guidelines. The combination of prednisone/hydroxychloroquine was used to treat the SLE patients. Twenty-five patients had spontaneous complete remission and were not treated (this data was added to Table 1). Even if complete remission was more frequent in non-nephrotic patients (46.2% vs. 33.3%) the difference failed to reach significance:

The follow-up seems short because the recruitment date ranged from June 2017 to April 2019 and we enrolled only newly diagnosed patients (prospective study).

The SLE patients were used as controls because they had a secondary membranous nephritis and some previous studies reported the presence of anti-PLA2R antibody in some SLE patients. Therefore, in order to better assess sensitivity and specificity of anti-PLA2R antibody measurement, we used SLE patients as the healthy group might be insufficient to obtain accurate results. We did not measure anti-PLA2R antibody in TIN patients because no previous study reported the presence of this autoantibody and glomeruli are not involved in TIN.

TIN patients were chosen as controls for mRNA analysis because they have theoretically non-injured glomeruli with a normal expression of PLA2R which is not the case for patients with membranous lupus nephritis. Moreover, only TIN patients (n=20) biopsies were available and we could not obtain enough usable biopsies for mRNA extraction from SLE patients.

Comment 6: We analyzed the rs4664308 SNP because it is considered as the most relevant polymorphism inside the PLA2R gene along with the rs2187668 SNP of HLA-DQA1. The strong linkage disequilibrium between rs4664308 and rs3749117 was described in European cohorts [Stanescu HC, Arcos-Burgos M, Medlar A, Bockenhauer D, Kottgen A, Dragomirescu L, et al. Risk HLA-DQA1 and PLA2R1 alleles in idiopathic membranous nephropathy. N Engl J Med 2011; 364(7): 616–26. doi: 10.1056/nejmoa1009742]. The linkage disequilibrium between two or more alleles varies ethnically and it would be interesting to analyze the rs3749117 in our patients in the future as indicated by reviewer #1.

Comment 7:

- At least 3 biopsy slices were used for RNA extraction for each patient. Reverse transcription for cDNA synthesis was made in one step together with the specific amplification of the target or the housekeeping genes. For cDNA synthesis we used the “Rotor-Gene SYBR Green RT-PCR master mix” (Qiagen) and random primers (48190-011, Invitrogen^TM^). The mix used for reverse transcription and cDNA amplification was as follow: 1) Rotor-Gene SYBR Green RT-PCR Master Mix (2X): 12.5 µl, 2) Rotor-Gene RT-Mix: 0.25 µl, 3) Random primers (150 ng/µl): 1.25 µl, 4) Extracted RNA (concentration: 2 to 4 ng/µl): 5 µl, 5) Specific primers Hs00234853_m1 (PLA2R) or Hs00922492_m1 (Podocin): 1.25 µl and 6): RNase free water: 4.75 µl. Thermal cycling was performed with an initial reverse transcription step at 55°C for 10 minutes, an activation step at 95°C for 5 minutes and then 40 cycles of denaturation at 95°C for 5 seconds and annealing/extension at 60°C for 10 seconds. We added these details to the methods section.

- We used podocin as reference gene in order to replicate the study of Hoxha E, et al (doi: 10.1038/ki.2012.209) in which the authors used the podocin as a housekeeping gene. we also compared the CT-values of podocin between TIN patients and PMN patients and there was no significant difference (p=0.284).

Thus, as shown there was no decrease of podocin expression in our PMN patients. Then we tested the correlation between CT-values of podocin and 24h-proteinuria, and there was no significant correlation, Spearman Rho = -0.112, p=0.362.

After that we tested the association between baseline (at the moment of kidney biopsy) nephrotic syndrome and podocin CT-values and again there was no significant association, p=0.865.

Since the CT-values did not vary between PMN and TIN patients and were not influenced by 24h-proteinuria or the presence of nephrotic syndrome we concluded that podocin was a good housekeeping gene in our study. Moreover, we have already compared the podocin and the RNA 18S Ct-values in 26 patients. There was a significant correlation between Ct-values of podocin and RNA 18S, Spearman Rho = 0.731, p=2.2E-5.

- We agree with reviewer #1 that immunostaining of biopsies and/or western blotting would be interesting in the future.

Comment 8: As recommended by reviewer #1 we added dot plots to figures 1, 2, 3 and 5. Additional box plot (1b) and dot plot (1c) were made to show separately and quantitatively anti-PLA2R Ab distribution and values in positive and negative PMN patients for anti-PLA2R Ab. We also added the threshold value (20 RU/ml) to figure 1 in box plots (1(a) and 1(c)) and in dot plots (1(b) and 1(d)). In figure 3, a dot plot (3b) showing PLA2R mRNA level in patients with positive anti-PLA2R Ab vs. negative vs. TIN patients. For figure 4, all PMN patients (positive and negative for anti-PLA2R Ab) were plotted for correlation with renal PLA2R level.

Comment 9: PLA2R mRNA levels and baseline 24h-proteinuria were not correlated, Spearman Rho = -0.069, p=0.577. This data was added to results section.

Anti-PLA2R Ab levels, and baseline 24h-proteinuria were not correlated, Spearman Rho = -0.080, p=0.515. We also added this data to results section.

Podocin CT values were not correlated to baseline 24h-proteinuria, Spearman Rho = -0.112, p=0.362.

Comment 10: Percentages for males vs. females are now compared vertically in Table 2. In table 1 there are no percentages comparing males vs. females but a descriptive data on sex-ratio for the PMN group. We indicated in tables 1, 2 and 4 when the parameters were measured (baseline or at diagnosis).

Comment 11: We analyzed the correlation between baseline 24h-proteinuria and PMN outcome and there was no significant association, p=0.422. We added this data to the results section.

A multivariable analysis was performed using the following covariables: baseline 24h-protéinuria, rs4664308 SNP, the presence of nephrotic syndrome at diagnosis, the presence of kidney failure at diagnosis, and the association between anti-PLA2R Ab (qualitatively and quantitatively) and PMN outcome remained statistically significant. This data was added to results section.

Also, another multivariable analysis was performed the association between PLA2R mRNA level and PMN outcome remained statistically significant. This data was added to results section.

Comment 12: Yes, previous study showed that age of PMN patients anti-PLA2R-positive vs. anti-PLA2R-negative was similar and we stated that our peculiar finding has not been noted in previous reports. We added the recommended citation by reviewer #1.

**Minor comments**

Comment 1: We replaced all “wild” with “wild-type” in the manuscript.

Comment 2: All “analytic results” have been deleted from the text.

Comment 3: It is the fold-change comparatively to a calibrator (biopsy from a kidney living donor). For each sample a relative expression of PLA2R to podocin was calculated. Then a fold-change for relative PLA2R expression was obtained from the calibrator.

Comment 4: We deleted or replaced most of “besides” in the text.

Comment 5: the legend for the Y axis in Figure 5 has been corrected.

**Reviewer** **#2:**

**Major comments**

Comment 1: TIN patients were chosen as controls for mRNA analysis because they have theoretically non-injured glomeruli with a normal expression of PLA2R which is not the case for patients with membranous lupus nephritis. Moreover, only TIN patients (n=20) biopsies were available and we could not obtain enough usable biopsies for mRNA extraction from SLE patients or kidney living donors.

Comment 2: Yes, all SLE patients had pure class V membranous lupus nephropathy. We specified this detail in subjects’ section.

Comment 3: PMN diagnosis was confirmed by **light microscopy** analysis of renal biopsies for all patients without staining for PLA2R or IgG4. We did not perform immunochemistry or direct immunofluorescence. We specified in the subjects’ section that PMN diagnosis was made on the basis of light microscopy. We also specified that all patients were screened for secondary membranous nephritis etiologies.

Comment 4: In this prospective study the anti-PLA2R Ab levels are baseline and in the same time at the time of biopsies. We specified it in the table 2.

Comment 5: In choosing a cut-off value of 239.95 RU/ml, the sensitivity and the specificity for non-remission prediction were respectively 100% and 87.5%. We updated this new data in the results and discussion sections. Thus, comparatively to the former cut-off value of 372.1 RU/ml, the sensitivity increased from 66.7% to 100% and the specificity decreased from 91.1% to 87.5%.

Comment 6: The lower mRNA level association with CKD can be explained the destruction of podocytes concomitant with the onset of renal failure. As the renal PLA2R expression is significantly correlated to anti-PLA2R Ab levels, and since this autoantibody level is associated with the non-remission outcome, this apparently paradoxical finding can be explained by the fact that lower mRNA levels induce less anti-PLA2R Ab and could be predictive of a better outcome despite its association with a baseline renal failure. This explanation has been added to the discussion section.

Comment 7: Since we screened SLE patients with secondary membranous nephritis we discussed our findings comparatively to previous studies in which a rare presence of anti-PLA2R Ab was reported. Consequently, we added possible explanations for the presence of this autoantibody in secondary membranous nephritides for which opinions differ, in fact some authors suggested that the presence of anti-PLA2R antibodies in patients with SMN might result from the co-incidental simultaneous development of PMN and a systemic disease, such as SLE or a malignancy. We felt that it is important to discuss the occurrence of anti-PLA2R Ab in secondary membranous nephritides even if it was negative in our SLE patients.

Comment 8: We changed ‘autoantibody’ to ‘immunological’ and as the immunological remission is well established, we changed ‘could be’ to ‘is’.

**Minor comments**

Comment 1: We corrected the errors mentioned by reviewer #2 and an academic English teacher has reviewed and corrected the manuscript.

Comment 2: We change p-values to <0.0001 when small.

Comment 3: We clarified that the rs4664308*G allele is protective.

Comment 4: We changed CKD classification into stage I to V in the table 1.

Yours sincerely

Tarak Dhaouadi
