# Supplementary material for: PLA2R antibody, PLA2R rs4664308 polymorphism and PLA2R mRNA levels in Tunisian patients with primary membranous nephritis
Source: PLoS One. 2020 Oct 1;15(10):e0240025. doi: 10.1371/journal.pone.0240025 (PMC7529277; doi:10.1371/journal.pone.0240025)
Supplement: Attachment — Submitted filename: Response to reviewers.doc [file pone.0240025.s005.doc]

**Letter to editor**

**List of changes**

**Revision point by point of the manuscript:** **PLA2R antibody, PLA2R rs4664308 polymorphism and PLA2R mRNA levels in Tunisian patients with primary membranous nephritis**

Dear editor,

As required, revision of our article “PLA2R antibody, PLA2R rs4664308 polymorphism and PLA2R mRNA levels in Tunisian patients with primary membranous nephritis” was made. All changes made are **in red color** in the “Revised manuscript with track changes”. We will provide here a point-by-point response to reviewers’ comments:

**Comments to the Author**

**Review comments to the author**

**Reviewer #1:**

**Major comments**

Comment 1: We added the fold-factors of PLA2R expression in non-remission vs. partial remission and vs. complete remission as requested by the reviewer #1.

Comment 2: We deleted all the sentences related to the etiology of anti-PLA2R Ab production.

Comment 3: As requested by reviewer #1 we replaced the link between PLA2R expression and autoimmune initiation by the correlation with anti-PLA2R production. The new sentence is: Some authors hypothesized that a renal overexpression of PLA2R could trigger the immune response and be correlated to anti-PLA2R Ab production. We also deleted the last sentence of the paragraph in which the PLA2R expression in lung could induce anti-PLA2R Ab.

Comment 4: We added this sentence in the introduction: Of note, in the study of Hoxha et al [8], the authors did not report any significant difference in PLA2R expression between patients with enhanced PLA2R staining and those with no enhanced staining.

In the discussion we added this sentence: Moreover, in the study of Hoxha et al [8], the compared groups included only MN patients while in our study the controls were TIN patients which could explain the discrepancy between the results. The following sentence explains why possibly an enhanced PLA2R staining is not necessarily correlated to PLA2R mRNA because of post-transcriptional increase.

Comment 5: We added this sentence while discussing PLA2R Ab levels correlation with PMN outcome: Even there is a limitation due to the presence of some non-nephrotic patients at baseline, …

We also added this sentence while discussing the association between PLA2R expression and PMN outcome: Nevertheless, the presence of some non-nephrotic patients at baseline remains a limitation in this regard.

Comment 6: As requested by reviewer #1, we added this sentence in the main text: Nevertheless, the linkage disequilibrium between two or more alleles varies ethnically and needs to be assessed in independent cohorts.

Comment 7:

We added this paragraph in the main text: We used podocin as a housekeeping gene in order to replicate the study of Hoxha E, et al [8]. Comparison of the CT-values of podocin between TIN and PMN patients showed no significant difference (*p*=0.284). Moreover, we tested the correlation between CT-values of podocin and 24h-proteinuria, and there was no significant correlation, Spearman Rho = -0.112, *p*=0.362. Likewise, podocin CT-values were not associated to nephrotic syndrome at the moment of kidney biopsy, *p*=0.865. Since the CT-values of podocin did not vary between PMN and TIN patients and were not influenced by 24h-proteinuria or the presence of nephrotic syndrome we concluded that podocin was a good housekeeping gene in our study. Moreover, comparison of podocin and the RNA 18S Ct-values in 26 PMN patients showed a significant correlation; Spearman Rho = 0.731, *p*=2.2E-5.

We discussed the discrepancy with the study of Hoxha E et al as requested in comment 4 and 7.

Comment 8: We added this sentence to Fig 4 legend: All PMN patients (positive and negative for anti-PLA2R Ab) were plotted for correlation with renal PLA2R level.

Comment 9: We already added this data “Podocin CT values were not correlated to baseline 24h-proteinuria, Spearman Rho = -0.112, p=0.362” as requested in comment 7 and comment 9

Comment 10: No request

Comment 11: We added this paragraph to the discussion section: In this study, 68 PMN patients were enrolled. Only 42 (61.8%) patients were nephrotic at the moment of diagnosis. Nevertheless, only 12 (17.6%) PMN patients evolved to a non-remission. Of note, PMN outcome was not influenced by baseline 24-hour proteinuria, *p*=0.422. This is an uncommon finding as proteinuria is generally recognized as a major prognostic factor [14]. This issue could be due to the generally good prognosis of our patients and the small size of our cohort.

We also specified in the results section the covariable used for the multivariable analysis.

Comment 12: No request

**Minor comments**

Comment 1: No request

Comment 2: No request

Comment 3: we added details of Fold-change calculation to fig 3 legend.

Comment 4: No request

Comment 5: the legend for the Y axis in Figure **4** has been corrected, the “anti-PLA2R Ab level” is on the left side now.

**Reviewer** **#2:**

No request

Yours sincerely

Tarak Dhaouadi
